# Establishing Sustainable Access to Quality Uterotonics in Kano, Lagos and Niger States—A Supply Chain Perspective

**DOI:** 10.1002/hpm.3910

**Published:** 2025-03-10

**Authors:** Eba Ajima, Chukwunonso Nwaokorie, Naanma Kangkum, Lola Ameyan, Obruche Sophia Ogefere, Eric Aigbogun, Valentine Amasiatu, Uchenna Igbokwe

**Affiliations:** ^1^ Solina Centre for International Development and Research (SCIDaR) Abuja Nigeria; ^2^ Clinton Health Access Initiative (CHAI) Boston Massachusetts USA

**Keywords:** heat‐stable Carbetocin, human‐centred design, market‐shaping, postpartum haemorrhage

## Abstract

**Background:**

The quality of oxytocin and misoprostol, the most widely used uterotonics for postpartum haemorrhage (PPH) management, suffer supply chain challenges and climactic susceptibilities.

**Aim:**

To describe a supply chain strengthening programme for introducing heat‐stable Carbetocin (HSC), to health facilities in Kano, Lagos and Niger states.

**Method:**

Human‐centred design (HCD) was employed to uncover uterotonics supply chain challenges and to identify priority interventions across a market‐shaping value chain to facilitate the rollout of HSC. Through a mixed‐methods approach and interviews with 203 stakeholders, challenges in the uterotonics supply chain and potential solutions were identified. A market‐shaping value chain was employed to map HSC introduction and rollout, focusing on key interventions. Before the project initiation, we established clear objectives including identifying barriers, introduction and rollout journey mapping of HSC.

**Results:**

Using HCD, HSC was successfully rolled out to over 87 health facilities. Employing the market‐shaping value chain, pivotal interventions were executed. These included policy updates (inclusion of HSC in the National and States Essential Medicines List), regulatory actions (registering Carbetocin with NAFDAC), financing strategies (co‐developing state roadmaps for sustainable procurements), supply management (reducing stock‐outs) and healthcare provider training on supply chain management practices. These efforts facilitated seamless integration of HSC into the states' supply chains.

**Conclusion:**

HCD and market‐shaping value chain approach were employed to introduce HSC in Nigeria. This study shows that integrating these approaches can enhance the availability and accessibility of essential medicines, offering potential replicability in similar health systems.

**Summary**

- Combining HCD and market‐shaping value chain frameworks improves medicine availability and sustainability.
- Continuous healthcare worker training is essential to maintain and enhance skills.
- Forecasting state's requirements is crucial for fair resource allocation and securing funding.
- Stakeholder collaboration is essential to introducing new medicines.

**Abbreviations**
AOPs: Annual Operational Plans
BMGF: Bill and Melinda Gates Foundation
DFID: Department for International Development
DMAs: Drug Management Agencies
DRF: Drug Revolving Fund
EML: Essential Medicines List
FMoH: Federal Ministry of Health
HCD: Human‐Centred Design
HSC: Heat Stable Carbetocin
IU: International Unit
LMIC: Low‐ and Middle‐income countries
LSS: Life‐Saving Skills
MMR: Maternal Mortality Ratio
NAFDAC: National Agency for Food & Drug Administration and Control
NDHS: Nigeria Demographic and Health Survey
NEML: National Essential Medicines List
PPH: Postpartum Haemorrhage
RACI: Responsible, Accountable, Consulted, Informed
SfM: Smiles for Mothers
SMoH: State Ministries of Health
USAID: United States Agency for International Development
WHO: World Health Organization

## 1 Introduction

### 1.1 Background

Nigeria bears a devastating maternal health crisis, suffering the highest number of maternal deaths globally and accounting for 28.5% of all such tragedies worldwide in 2020. In addition, the country's alarming maternal mortality ratio (MMR) of 1047 per 100,000 live births, ranking as the third‐highest globally, underscores the urgency of addressing this critical public health issue [1]. This national average, however, masks significant regional disparities. Northern states such as Kano and Niger report MMR values of 1625 and 1463 per 100,000 live births respectively, far exceeding the national average by 55% and 40%. These figures starkly contrast with Lagos, a southern state, where the MMR stands at 555, nearly 47% below the national average [2].

Studies indicate that postpartum haemorrhage (PPH) is a leading cause of maternal mortality in Nigeria, with rates ranging from 26% to 27.7% across different regions [3, 4]. Uterine atony is responsible for the majority of cases, and active management of the third stage of labour (AMTSL) using uterotonics, such as oxytocin and misoprostol, can reduce its occurrence and complications [5, 6]. However, challenges in low‐and middle‐income countries (LMICs) like Nigeria, such as inadequate cold‐chain infrastructure and unreliable electricity, impair proper storage of oxytocin between 2°C and 8°C [7, 8]. Misoprostol, an alternative uterotonic, also has a proclivity for degradation due to the climatic conditions of many LMICs [8].

A market survey by Nigeria's National Agency for Food and Drug Administration and Control (NAFDAC) further quantified these challenges. During this survey, 74.2% of the oxytocin injections and 33.7% misoprostol tablets sampled across the six geopolitical zones in the country failed the required assay standards [8], highlighting the poor quality of uterotonics available in health facilities. This issue is further exacerbated by ignorance among healthcare providers regarding the appropriate storage conditions for uterotonics [8, 9]. A study on oxytocin usage by healthcare providers in 12 Nigerian states found that only 52% of the healthcare providers were aware of its storage needs of 2°C–8°C, and 51% used a higher or excessive dose than recommended for PPH management [9].

In response to these challenges, the WHO updated its recommendations in 2018 to include Carbetocin (heat‐stable formulation) as a uterotonic option for PPH prevention, especially in settings where oxytocin quality cannot be guaranteed [10]. This was in light of evidence from the CHAMPION trial, a randomized clinical trial that demonstrated Carbetocin's non‐inferiority to oxytocin for PPH prevention during the third stage of labour. The trial also positioned Carbetocin as the highest‐ranked single uterotonic agent for the prevention of blood loss. In addition, a 2018 network meta‐analysis further underscored the efficacy of heat‐stable Carbetocin in preventing blood loss ≥ 500 mL and potentially reducing the need for additional uterotonics [11].

These recommendations also emphasised the importance of training healthcare workers to ensure the safe and correct use of these medicines at all levels of the health system [10], as limited knowledge on the proper use of uterotonics and their storage requirements contributes to poor quality and can cause harm to patients [9, 12, 13].

### 1.2 Study Rationale

The Smiles for Mothers (SfM) programme, funded by MSD for Mothers is a collaborative initiative launched in 2020, comprising Solina Centre for International Development and Research (SCIDaR), the Clinton Health Access Initiative (CHAI) and the Co‐Creation Hub (CcHUB), focused on reducing maternal mortality in Nigeria, with a particular emphasis on addressing PPH. Following the guideline updates, the SfM programme supported the Federal Ministry of Health (FMoH) and the state governments of Kano, Lagos and Niger to design and implement innovations for rolling out the 2018 WHO recommendations on uterotonics. The objectives were to reduce PPH‐related disabilities and deaths and strengthen the supply chain of existing and new uterotonics in the states, thereby addressing the lack of availability of quality uterotonics at service points. By reducing PPH‐related death, the study will contribute to the Sustainable Development Goal (SDG) Target 3.1, which is aimed at reducing the global maternal mortality ratio to less than 70 per 100,000 live births. Improving access to quality medicines for the management of PPH, a leading cause of maternal deaths, can enhance maternal outcomes, and bring Nigeria closer to achieving this SDG target.

However, various challenges were identified across different components of the uterotonics supply chain. These include poor quantification and data visibility, inappropriate storage, ineffective procurement practices and inefficient inventory management strategies, which impair new product introduction and maternal healthcare quality, thereby contributing to high maternal mortality [7, 14, 15, 16, 17]. Inadequate funding of public health facilities by Federal and State governments also stands as a root cause of the unavailability of essential medicines, including uterotonics [18].

At the health facility level, delayed reimbursements to state governments for supplied essential medicines create supply bottlenecks [7]. Poor data collection, quality, and use also limit the ability to make accurate forecasts [19]. Other challenges commonly encountered include the procurement of substandard medicines, in part due to inadequate funding sources, and frequent stockouts of essential medicines due to inefficient inventory management systems [14, 19, 20]. These limitations highlight the need for a strategy to enhance medicine supply chain systems.

Human‐centered design (HCD) is a user‐centric process that involves end users in defining the problem and ideating, prototyping and testing potential solutions [21]. HCD has been applied extensively through interviews and focus group discussions with key stakeholders to understand their experiences and challenges in accessing and distributing medicines. The process can be translated to developing potential solutions that can be adopted to improve the uterotonics supply chain, such as enhancing transportation infrastructure or providing training for health workers on uterotonic use [22]. These solutions will be subsequently prototyped and tested in the field, with feedback from end‐users used to refine interventions [21, 22].

Meanwhile, a framework that has been applied successfully to improve access to essential health commodities in low‐resource settings is the market‐shaping value chain [23, 24, 25]. For a new medicine, this framework comprises all the actors involved in the product‐introduction process, including pharmaceutical companies, regulators, wholesalers, distributors, procurement agents, administrators and healthcare providers, to ensure a reliable supply of commodities [26].

Market‐shaping interventions have been applied at different points in the value chain to improve access to essential medicines, such as artemisinin‐based combination therapies (ACTs) and antiretroviral therapy (ARTs) for people living with HIV/AIDS in LMICs [27]. These strategies have also been employed in addressing specific barriers to achieve lower price negotiations with manufacturers, create demand for commodities through public awareness campaigns, and ensure a reliable supply of health commodities [18, 26, 27].

Based on the successes of these strategies in enhancing healthcare delivery and medicine access, this paper sought to describe the interventions implemented in Kano, Lagos and Niger States by the Smiles for Mothers program. The primary aims were to strengthen the supply chain system for uterotonics and facilitate the introduction of heat‐stable Carbetocin, specific to PPH prevention, using the human‐centered design and market‐shaping value chain approaches.

## 2 Methods

### 2.1 Study Setting

The research was conducted in primary and secondary health facilities in Kano, Lagos and Niger States. Kano State stands as one of Nigeria's most densely populated states, with urban centres enjoying comparatively better healthcare facilities than rural areas. The state has 1486 facilities spread across its 44 Local Government Areas (LGAs), with 1274 publicly owned. Of these public facilities, 1224 (96%) are primary healthcare facilities, 48 are secondary and the remaining 2 are tertiary [28].

Meanwhile, Lagos has more advanced healthcare infrastructure compared to many other states, with several tertiary hospitals and a vast number of secondary and primary healthcare facilities. The state boasts of 2206 operational health facilities of which 415 are publicly owned. Of these 372 are primary facilities, 39 are secondary and 4 are tertiary [28].

Niger State also has varying healthcare infrastructure, with more robust resources in urban areas, limited facilities in rural areas and very poor access in certain regions. Despite having a spread of 1565 healthcare facilities across 25 local government areas, the state suffers a critical healthcare challenge. This is because out of 1498 publicly and privately owned primary healthcare facilities, only 274 (20%) of them are operational. The remaining facility levels comprise 64 secondary and 3 tertiary facilities, where 28 secondary and two tertiary facilities are publicly owned [28].

These locations were chosen as study sites due to their high maternal mortality ratios, their existence as representatives of their geopolitical zones, and the propensity to achieve rapid and sustainable scale for the rollout of HSC for PPH prevention.

### 2.2 Sample Size

The project implementation targeted a total of 87 public healthcare facilities in Kano, Lagos and Niger states. This comprised 3 high‐volume primary healthcare facilities (PHFs) in each state, along with 74 secondary health facilities (SHFs)—31 in Kano, 22 in Lagos, and 21 in Niger‐ and 4 tertiary facilities—1 each in Kano and Lagos, and 2 in Niger. The health facilities were selected due to their high volume of deliveries and willingness to participate in the research. The research population was 807, comprising 10 health workers from secondary and tertiary facilities (THFs) and 3 from primary health facilities. Ultimately, a total of 770 health workers participated in the training. The calculation is detailed below: 87 Target number of facilities: 4 tertiary facilities, 74 secondary health facilities and 9 primary healthcare facilities. Personnel per facility targeted for training by the SfM programme: 4 Doctors/ObGyns, 6 Nurses/Midwives. Total population size: 10 healthcare workers × (4 THFs + 74 SHFs) + 3 healthcare workers × 9 PHFs = 807 health workers.

On the other hand, 203 participants across the three states were involved in the qualitative study. This comprised 97 healthcare workers, traditional birth attendants (TBAs), and agents from the Community Health Influencers Promoters and Services (CHIPS) Programme, as well as 26 government stakeholders, and 80 pregnant women and nursing mothers, as shown in Table 1.

**TABLE 1:** Summary of interviews conducted by state.

State	Facilities engaged	Health workers, TBAs, CHIPS agents	Government stakeholders	Pregnant women and nursing mothers
Kano	12	33	8	28
Lagos	10	29	8	26
Niger	11	35	10	26
Total	33	97	26	80

### 2.3 Study Design

This study employed a human‐centred design (HCD) approach to explore the challenges associated with the supply chain for uterotonics in Kano, Lagos and Niger states, and to propose solutions to overcome these challenges. It involved a review of existing literature on the topic, alongside a four‐step HCD approach, comprising co‐research, co‐design, co‐refinement and implementation.

During the co‐research phase, interviews were conducted with 203 participants across the three states. These interviews enabled the programme to analyse and identify priority challenges in the uterotonics supply chain, as well as develop appropriate interventions geared towards effective system strengthening.

**FIGURE 1:**
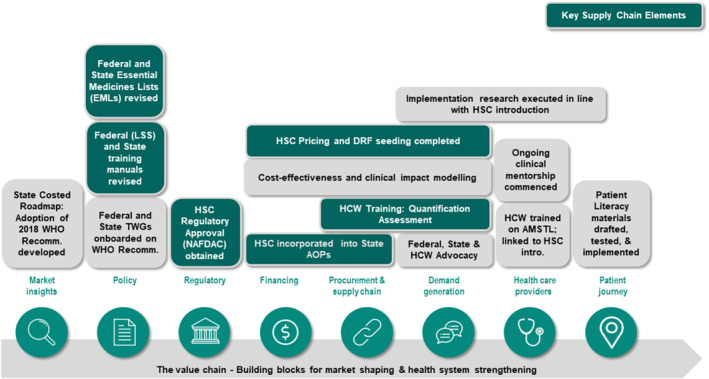
The market‐shaping value chain framework.

Subsequently, the programme adopted a market‐shaping value chain framework in the co‐design phase to map the product journey to roll out the 2018 WHO recommendations on uterotonics to prevent PPH and for the introduction of heat‐stable Carbetocin (see Figure 1). The market‐shaping value‐chain approach aimed to address the challenges in uterotonics supply chain management, including policy, regulation, financing, procurement, storage and distribution. This approach involved collaboration with stakeholders at different levels of the supply chain, including pharmaceutical companies, regulators, distributors, procurement agents, administrators, pharmacists and other healthcare providers.

The research findings were used to inform the co‐refinement process, continuously refining interventions based on feedback from relevant stakeholders across the supply‐chain value chain. This approach also included the development of training programs and job aids to enhance the healthcare workforce's capacity in PPH management broadly.

Finally, the implementation phase targeted a total of 87 public healthcare facilities in the three states. This comprised 3 high‐volume primary healthcare facilities in each state, along with 74 secondary health facilities—31 in Kano, 22 in Lagos, and 21 in Niger‐ and 4 tertiary facilities—1 each in Kano and Lagos, and 2 in Niger. The steps in the implementation of the market‐shaping value chain comprised:

- *Gaining market insights*: The consortium leveraged the expertise of eight human‐centred design specialists to gain insights into the needs and desires of administrators, pharmacists and healthcare providers, especially regarding PPH management and uterotonics. Based on these insights and the need for reliable quality uterotonics, a costed plan for the introduction of HSC in Kano, Lagos and Niger was developed in partnership with the state champions.
- *Obtaining regulatory approval*: New drugs introduced into the Nigerian market must undergo a regulatory approval process conducted by the National Agency for Food and Drug Administration and Control (NAFDAC). This process involves evaluating the drug's safety, efficacy and quality, and assessing its potential impact on public health. NAFDAC may also consider factors such as the cost and availability of the drug before granting approval for its use. Consequently, the consortium supported the IDA Foundation to facilitate NAFDAC's regulatory decision on HSC use in Nigeria.
- *Updating relevant national guidelines and local normative policies* to provide the necessary foundation for introduction. After a review of the existing national guidelines and policies related to the use of uterotonics in Nigeria, the SfM Consortium consulted key stakeholders to advocate for updating the national guidelines, namely the National and State Essential Medicines Lists (EML) and Life‐Saving Skills (LSS) training manuals. This resulted in the review of the 2018 WHO recommendation on uterotonics for PPH prevention, facilitating evidence‐based updates to the current basket of uterotonics and the inclusion of HSC into these policies.
- *Securing sustainable financing mechanisms* to ensure equitable access to appropriate quantities of uterotonics, including HSC. The project conducted a comprehensive needs assessment by estimating the quantity of uterotonics, including HSC, required by each state as well as the associated costs. Following a review of potential funding sources based on their adequacy and availability for the procurement of uterotonics, stakeholders identified the states' Drug Revolving Fund (DRF) as the most sustainable financing mechanism for uterotonics, particularly for the introduction of HSC into the programme states. The programme supported the states to conduct learning exchange sessions on sustainable financing for uterotonic procurement, which led to the development of state recommendations and roadmaps to optimise the procurement process. Furthermore, the states were provided with an initial stock of HSC for insertion into the DRF mechanism to kick‐start availability and revenue generation and, with demand, continued availability of the medication in the states.
- *Establishing reliable mechanisms for procurement, storage and efficient distribution* of uterotonics including HSC to health facilities providing obstetric services. SfM conducted an analysis of the procurement, storage and distribution of essential medicines in the states to identify opportunities for improvements and aligned with relevant state stakeholders to integrate HSC into existing state uterotonic supply chain management processes to enhance the sustainable supply of uterotonics to the last mile.
- *Generating demand for heat‐stable Carbetocin through advocacy to relevant policymakers, health managers and clinicians:* The programme aimed to increase the demand for HSC for PPH prevention based on its evidence‐base and normative policy inclusion. To achieve this, the programme implemented a communication plan comprising three main components: advocacy, stakeholder communication and programme messaging. Advocacy efforts focused on raising awareness among healthcare providers, policymakers and community leaders about the importance of preventing PPH, the concerns around current uterotonic quality and the availability of HSC as an alternative for PPH prevention. The stakeholder communication plan identified key stakeholders, their roles, and the best approach for communicating with them. The RACI (responsible, accountable, consulted and informed) responsibility assignment framework was used to categorise stakeholders based on their involvement in the programme subthemes. Two key messages that aligned with the project goals were developed: the adoption of the 2018 WHO recommendations for PPH prevention and the use of HCD in public health interventions. These messages were disseminated to target audiences within defined timelines.
- *Training of healthcare workers*: SfM also aimed to ensure that healthcare workers from these three states were equipped with the necessary skills to use uterotonics during labour and delivery, including HSC for PPH prevention. This was achieved by training 772 doctors, nurses and midwives on the most up‐to‐date knowledge of emergency obstetric and newborn care (EmONC), active management of the third stage of labour (AMTSL), pharmacovigilance and supply chain management (Table 2). In addition, 136 pharmacists received training on supply chain management to improve the availability of uterotonics. The capacity‐building intervention used a blend of in‐class sessions and on‐the‐job coaching to ensure that participants acquired knowledge and applied it to real‐life situations. In‐class sessions were conducted centrally and in clusters, where participants were trained on selected topics using various training techniques such as lectures, demonstrations and group and individual exercises. The second approach involved on‐the‐job coaching in which field mentors observed participants as they conducted their job routines and provided feedback. Materials such as coaching flow maps and ODK (open data kit) data collection tools were used to assist the participants as required.

## 3 Results

The study identified some priority challenges which include the lack of training materials in formats tailored to each health worker cadre, the uncertainty of uterotonic potency prior to administration, leading to practices of increasing dosage or combining uterotonics against WHO recommendations, and HCWs having limited awareness of risk factors associated with PPH and uterotonics.

**TABLE 2:** Breakdown of the training participants by state.

State	Number of trained HCWs	Number of trained pharmacists
Kano	311	51
Lagos	241	56
Niger	220	29
Total	772	136

Some quotes from the KIIs are presented below:

Because of the low potency of oxytocin due to the lack of fridges, double doses of this medicine is usually recommended.

—TBA, Kano State

We use two drugs here: oxytocin and misoprostol; the oxytocin we have here is not very potent, so we combine it with misoprostol.

—Doctor, Kano State

PowerPoint presentations … videos … practical demonstrations on the active management of third stage of labour, and showing how to use antishock garments will go a long way.

—Doctor, Niger State

### 3.1 Market Insights: Developed Costed Roadmaps for Implementation of the 2018 WHO Recommendations for Uterotonics

**FIGURE 2:**
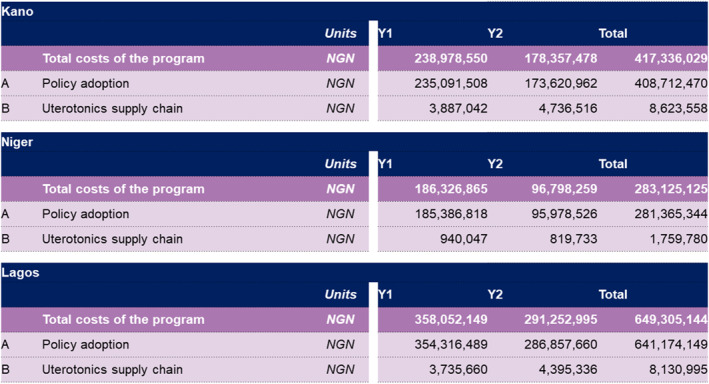
Costed roadmaps which estimate incremental costs associated with the rollout and adoption of the 2018 WHO recommendations for the prevention of postpartum haemorrhage in Kano, Lagos and Niger.

Using HCD, the programme co‐designed 17 solutions to support the appropriate use of uterotonics in preventing PPH and strengthening the supply chain for these medicines. These solutions are summarised in Table 3. Subsequently, the programme developed roadmaps outlining the activities proposed by each state with their corresponding costs (see Figure 2).

**TABLE 3:** The set of 17 solutions co‐designed with relevant state stakeholders to support appropriate uterotonic use for PPH management.

Opportunity space	Key recommendation	Solutions
1. How to design a comprehensive publication and dissemination strategy for the WHO recommendations?	Apply a systematic approach for disseminating information from the guidelines that is differentiated by audience type and tailored to the intended audience to support optimal understanding and retention of the new guidelines	Develop an approach for disseminating guidelines in public hospitals/clinics
		Develop an approach for disseminating guidelines in private hospitals/clinics
		Develop an approach for disseminating guidelines in non‐facility delivery places for example, TBAs, FBO
		Define a consistent and standardized approach for monitoring and evaluating the implementation/application of the guideline
		Leverage digital platforms to disseminate information on changes
2. How to enhance the level of awareness and adoption among skilled healthcare workers of the guidelines to increase the use of appropriate uterotonics	Support state‐sponsored training and step‐down training for each cadre of skilled HCWs and tailor curriculum and training materials for all types of HCWs	Differentiate training curriculum for skilled HCWs in different HF levels when integrating new guideline content into existing training programs
		Develop tailored training materials for skilled HCWs for example, doctors, nurses, midwives
		Develop tailored training materials for pharmacists and pharmacy technicians
		Provide training tailored to the learning needs of semi‐skilled HCWs to ensure better adoption of guidelines
		Improve step‐down training in healthcare facilities for both skilled and semi‐skilled HCWs (at least quarterly)
3. How to enhance the level of awareness and adoption among unskilled healthcare workers of guidelines to increase the use of appropriate uterotonics for PPH prevention in all deliveries	Upskill TBAs through training to identify danger signs of PPH, strengthen partnerships and linkages of TBAs with formal health system, and eventually find new roles for TBAs within the formal health system	Upskill TBAs to make sure they educate on PPH prevention and encourage pregnant women to attend ANC
		Strengthen linkages between TBAs, the community and HFs to improve the rate of facility‐based deliveries
		Find new roles for TBAs within the formal health system
4. How to leverage community institutions to cascade health recommendations to the community to ensure families are better aware of the risks of PPH, the availability of prevention mechanisms and the benefits of giving birth in a facility	Leverage new and existing community institutions to raise awareness about PPH: Risk factors, availability of prevention, the benefits of giving birth in a facility and actions to take in the event of PPH	Empower PPH ambassadors (e.g., mothers, influencers, celebrities) to advocate for PPH prevention and convince women to go to the facility to deliver
		Leverage every relevant health‐related touchpoint with mothers to communicate about PPH
		Leverage non‐health‐related touchpoints to communicate about PPH
		Beyond awareness, empower communities to take action in case of PPH

### 3.2 Securing Sustainable Financing

**FIGURE 3:**
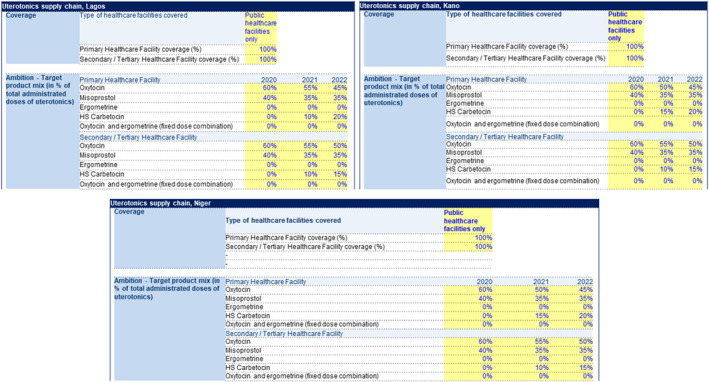
Table showing the phased introduction of heat‐stable Carbetocin in Lagos as a function of the product mix for other uterotonics.

The three focus states operate DRF to supply essential medicines to over 800 healthcare facilities. Therefore, SfM worked to optimise this scheme by providing an initial seed stock of 69,000 HSC ampoules to generate funds for subsequent orders. The programme also evaluated the potential uterotonic mix for each state following the introduction of HSC (see Table 4, Figure 3). In addition, it worked towards building an investment case for the introduction of the new medication, presenting an argument for investing in maternal health because of its potential impact on health outcomes. This was achieved through the collaboration of health economists, clinicians and maternal health programme managers to obtain early buy‐ins for this study.

**TABLE 4:** Model showing annual uterotonic needs of the states over a 6‐year period.

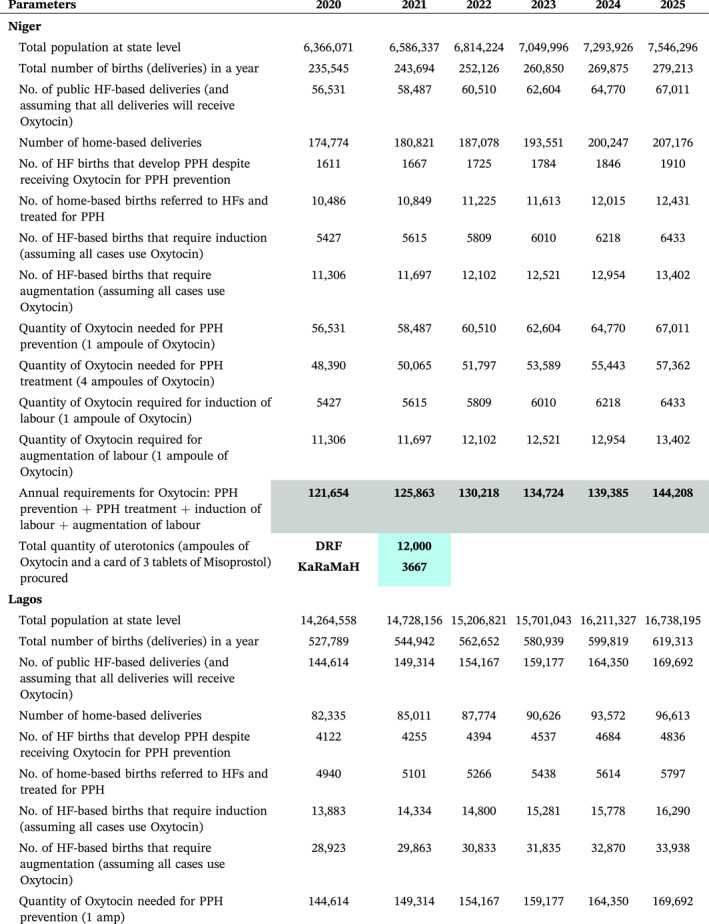
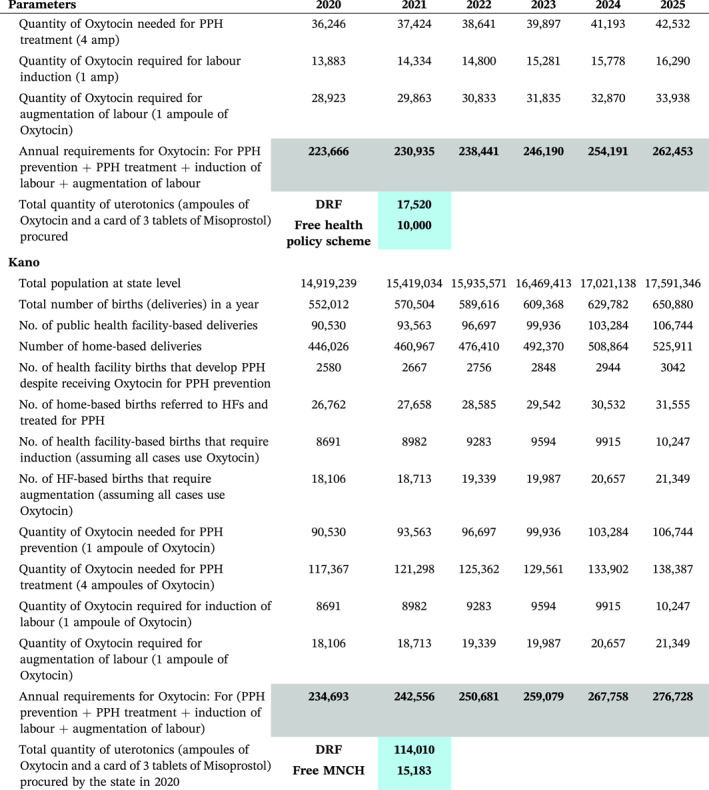

Following the application of various DRF markups, the cost of HSC was found to vary across the states, and the markups were captured in DRF operational guidelines. With this clarity on the potential cost of HSC to the states, the Consortium and IDA Foundation engaged the three states to discuss and align on the price based on the volatility of the Naira‐to‐Dollar exchange rate and potential additional logistics and storage service charges when states commenced procurement.

### 3.3 Procurement and Supply Chain: Establishing Reliable Mechanisms for Procurement, Storage and Efficient Distribution

In the programme states, the Drugs Management Agencies (DMAs) play a central role in overseeing the flow of commodities from manufacturers to service delivery points.

To support this system, the SfM programme imported and distributed the seeded ampoules for training and programme implementation in Kano, Lagos and Niger for clinical training and pilot implementation. The clinical training involved mentoring health workers on the use of HSC through implementation research, and free samples were distributed to high‐volume secondary health facilities to document acceptability and their utilization patterns for the drug.

### 3.4 Demand Generation: Generating Demand for Heat‐Stable Carbetocin Through Advocacy

The consortium promoted knowledge sharing across programme and non‐programme states through various platforms, including newsletter publications, webinars and conferences. During programme implementation, a comprehensive analysis of the programme's mailing list, which encompassed stakeholders from all 36 Nigerian states plus the Federal Capital Territory, and international locations, revealed that 739 unique stakeholders actively engaged with the programme's communications. This was primarily determined by the number of persons who opened the programme's newsletter, of which 9 editions were disseminated. Notably, the programme's influence extended beyond its focus states, with confirmed engagement from recipients in 32 non‐programme states. This broad geographical reach was verified through a meticulous analysis that identified at least one recipient from each of these states who meaningfully interacted with the newsletter content.

The programme also participated in five webinars, hosted by the Reproductive Health Supply Coalition (RHSC), The Core Nigeria, The Curve, Nigeria Health Watch and the Smiles for Mothers programme, during which the application of HCD and programme innovations were discussed. The team developed patient literacy materials in collaboration with relevant state stakeholders and reached 5643 women with the materials, across the three programme states. These patient literacy materials were designed to generate demand for health facility services in a broader context, extending beyond the specific focus on heat‐stable Carbetocin.

SfM also hosted two (2) learning exchange workshops for stakeholders across the three states. The first was conducted to develop a state‐specific roadmap for sustainable financing of maternal health commodities, including uterotonics. The second learning exchange workshop was conducted with the aim of strengthening the postpartum haemorrhage (PPH) response and maternal, perinatal and child death surveillance and response (MPCDSR) structures across programme states.

### 3.5 Healthcare Workers: Training and Capacity Building

Overall, there was an increase in the knowledge of healthcare workers in all the states. In Kano, knowledge increased from 58% to 74% among doctors, and 39%–67% among nurses and midwives. For Lagos State, an increase from 57% to 74% for doctors and 53%–68% for nurses and midwives was experienced. In Niger, the overall knowledge improved from 51% to 65.9%. Beyond the classroom training, the programme introduced a mentoring initiative, with field officers conducting bi‐weekly visits to health facilities. During these visits, they observed and supported pharmacists and technicians in areas of identified gaps, concentrating on inventory management, data management and pharmacovigilance. These mentoring sessions resulted in a remarkable 50% reduction in uterotonic stockout rates in 12 health facilities with about 31 supported facilities recording no stockouts, compared to a similar duration before the initiation of mentoring sessions.

## 4 Discussion

### 4.1 Market Insights

The Smiles for Mothers Programme was launched with the primary objective of reducing maternal mortality caused by postpartum haemorrhage (PPH) in Kano, Lagos and Niger States. It commenced with using human‐centred design (HCD) to co‐develop sustainable solutions with relevant stakeholders to mitigate the burden of PPH in these states. In addition, the programme combined the market‐shaping and value chain approaches to identify all relevant stakeholders or actors and the appropriate strategies to ensure success at all levels.

This enabled the programme to pinpoint the core challenges experienced by the states and develop strategies to address them. One of these strategies involved developing a costed roadmap outlining the incremental costs associated with adopting and rolling out the WHO recommendations for uterotonics for PPH prevention, tailored to the local contexts of each state as proposed by the respective stakeholders (see Figure 2).

### 4.2 Updating Relevant National Guidelines and Local Normative Policies

The policy pathway focused on creating a conducive policy environment for the rollout of this programme, ensuring all relevant policy documents were updated and aligned with the WHO 2018 recommendations on uterotonics for PPH prevention. The provider pathway was centred on equipping healthcare workers with the necessary skills and knowledge to deliver safe and effective PPH care. Finally, the client journey involves generating patient demand for skilled delivery and healthcare services.

The relevant policies for the successful implementation of this programme comprised the national and states' essential medicines lists (EML) and the lifesaving skills (LSS) manual. Since Nigeria runs a decentralised healthcare system, there is a need for states to adapt and domesticate their policies to align with the national provisions. The policy review process begins with engaging relevant state stakeholders to obtain state approval, followed by securing funding, convening potential participants and coordinators to conduct the review, and then validating the reviewed documents.

The major challenges experienced by many states involve delays in implementing these reviews particularly due to funding constraints and lack of political will. This was evident in Lagos, where the most recent EML update occurred in 2012. This necessitated advocacy visits to relevant stakeholders to address bottlenecks and obtain approval before the programme provided financial and technical support in facilitating the required policy changes. Consequently, the SfM programme supported the Kano, Niger and Lagos States' Ministries of Health to update and disseminate their state EMLs following the national review. The programme also extended its support for the review and validation of the national LSS manual.

### 4.3 Securing Sustainable Financing

The DRF is a self‐sustaining financing scheme designed to maintain supply of medicines and commodities by reinvesting generated revenue, thereby minimising costs for the patients and increasing income for the state. Seeding to the drug revolving funds (DRF) aimed to sustainably roll out HSC and generate funds for additional procurement. Kano State had the highest use due to the state's high birth rate, while Niger had high use during deliveries but lower in‐facility births. Lagos had a relatively low consumption because the drug was mainly administered during cesarean sections and high‐risk deliveries based on a past history of use of the commercial brand (Pabal). The implementation proved that the seed stock was vital for the success of HSC adoption by states and ensured subsequent orders through the DRFs.

Furthermore, due to the SfM programme's intervention in reviewing state procurement processes and suggesting improvement recommendations, the Kano State Drugs Management and Consumables Supply Agency initiated the procurement of a superior quality brand of Oxytocin for its DRF. This has resulted in enhanced service delivery and increased satisfaction among health workers.

Since the implementation of these interventions, the programme has recorded positive results, as all three states have placed orders for additional stock of HSC following utilization of the stock seeded to their DRFs.

### 4.4 Procurement and Supply Chain: Establishing Reliable Mechanisms for Procurement, Storage and Efficient Distribution

Mapping the maternal health commodity supply chain revealed a complex network of stakeholders and mechanisms for coordinating the procurement, storage and distribution of essential medicines (see Figure 4a–c). A major drawback of the effective drug supply flow is the unavailability of cold‐chain equipment and/or unstable power supplies for the appropriate storage of heat‐sensitive commodities, and the elimination of this requirement amplified HSC's acceptability.

FIGURE 4(a) Overview of the supply chain for maternal health commodities in Lagos. (b) Overview of the supply chain for maternal health commodities in Kano. (c) Overview of the supply chain for maternal health commodities in Niger.

**Figure:**
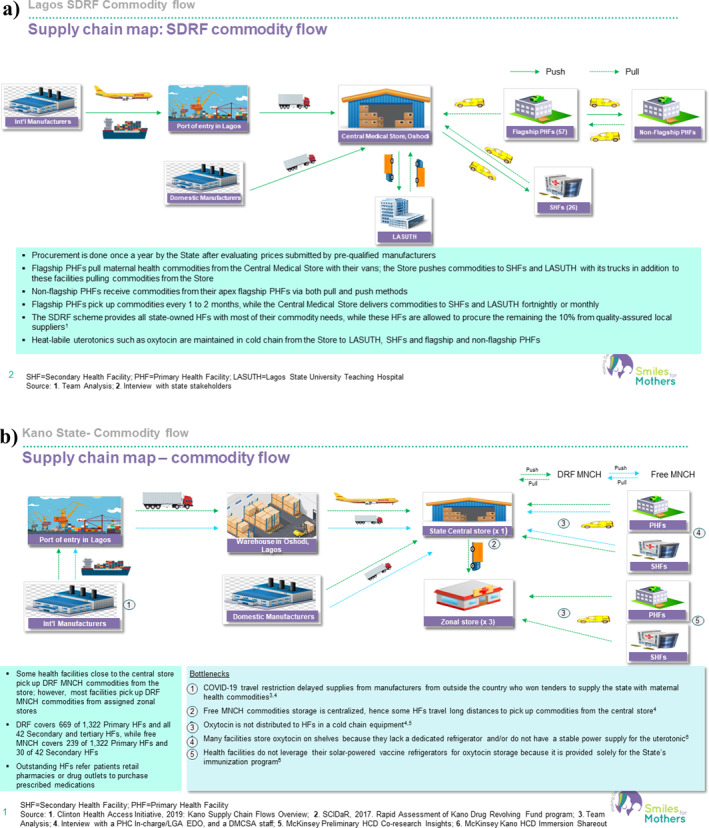

**Figure:**
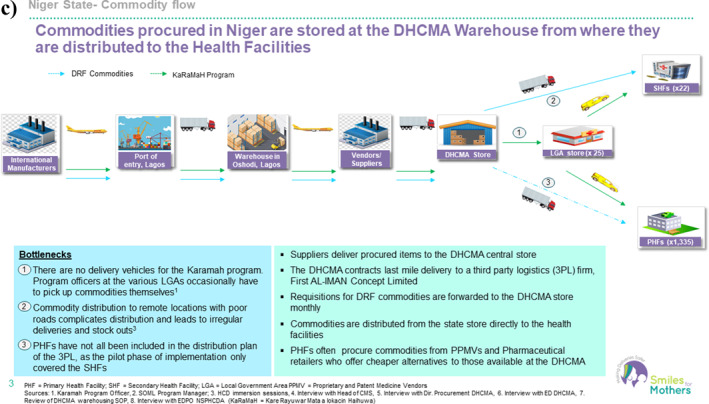

In the program states, the Drug Management Agencies (DMAs) play a central role in overseeing the flow of commodities from manufacturers to service delivery points. The DMA committees and working groups are composed of personnel from various state ministries, departments, parastatals and partner organizations to ensure efficient coordination of commodity procurement, logistics, storage and data management.

For an effective flow, maternal health commodities are procured by DMAs (for Lagos, the Central Medical Store) from pre‐qualified manufacturers and distributed to public health facilities through a pull system. Free MNCH programs funded by states provide maternal health commodities to patients at no cost, whereas DRFs enable states and health facilities to sell drugs to clients at a predetermined markup to support the procurement of more drugs.

### 4.5 Healthcare Workers: Training and Capacity Building

The training of health workers offered a rare forum for participants to interact in a collegiate environment, particularly in Kano and Niger, which may have contributed to the positive reaction to the training, as central capacity building activities for pharmacists are rare in these states. Overall, the programme was successful in meeting participants' expectations and resulted in significant knowledge gain. This signified the importance of consistent training programs to ensure the skills of health workers are sufficient to provide quality healthcare.

In addition, the SfM programme implemented supply chain focused training for pharmacists and pharmacy technicians in the focus states on the appropriate storage and supply management of uterotonics in the health system supply chain. The training modules included forecasting methodologies, quantification, storage practices, warehousing, data management, distribution and pharmacovigilance, with significant knowledge gains observed in each module across states.

Training sessions were required to refresh and update participants' knowledge of the supply chain workstream, with pharmacovigilance being an entirely new module for most participants. The results (see Table 5) indicates that the quantification and pharmacovigilance modules showed the most significant improvement in participant scores in Kano. The success of the training programme demonstrates the positive impact of capacity building for pharmacists and highlights the need for ongoing training and support to maintain and improve knowledge and skills in the field.

**TABLE 5:** Results of pharmacists and logisticians capacity building.

	State	Modules	0	1	2	3	4	5	No score
Pre‐test	Lagos (*n* = 56)	Quantification	4	1	15	18	12	5	1
		Commodity storage	0	1	3	27	20	4	1
		Distribution	0	1	2	10	22	20	1
		Data management	0	1	6	5	20	23	1
		Pharmacovigilance	0	5	13	16	16	5	1
Post‐test		Quantification	0	1	7	10	13	22	3
		Commodity storage	0	0	0	2	14	37	3
		Distribution	0	0	0	4	15	34	3
		Data management	0	0	3	4	16	30	3
		Pharmacovigilance	0	0	2	10	27	14	3
Pre‐test	Niger (*n* = 28)	Quantification	2	2	8	9	5	2	0
		Commodity storage	1	1	7	6	10	3	0
		Distribution	1	0	3	8	11	5	0
		Data management	2	2	3	3	6	12	0
		Pharmacovigilance	2	0	5	8	11	2	0
Post‐test		Quantification	0	1	2	3	11	11	0
		Commodity storage	0	2	0	3	9	14	0
		Distribution	0	1	1	4	11	11	0
		Data management	0	0	0	6	3	19	0
		Pharmacovigilance	0	1	2	3	13	9	0
Pre‐test	Kano (*n* = 51)	Quantification	2	12	11	17	8	1	0
		Commodity storage	6	7	14	10	12	2	0
		Distribution	1	6	10	14	14	6	0
		Data management	3	6	9	10	19	4	0
		Pharmacovigilance	9	11	9	11	6	5	0
Post‐test		Quantification	0	0	4	16	20	11	0
		Commodity storage	0	0	3	16	19	13	0
		Distribution	0	0	1	10	29	11	0
		Data management	0	0	1	4	29	17	0
		Pharmacovigilance	0	0	0	3	27	21	0

Hence, other programs can build on these gains to establish pharmacovigilance training and strengthen supply chain interventions in the region.

## 5 Conclusion

The Smiles for Mothers programme successfully introduced a new uterotonic, heat‐stable Carbetocin, into three states in Nigeria by integrating the market‐shaping value chain into the human‐centred design (HCD) approach. This process involved a combination of strategies, such as obtaining regulatory approval, updating policy documents and training healthcare workers on HSC use, EmONC and supply chain management to establish an efficient and sustainable uterotonic supply chain. The findings from this study demonstrate that combining HCD and market‐shaping value chain frameworks can improve the availability, accessibility and sustainability of medicines in health facilities by ensuring that key strategies are carried out. In addition, the lessons learnt can be applied by other programs or countries when introducing new health products. These strategies can potentially promote the sustainable supply of essential medicines in Nigeria, improve the quality of maternal care, and reduce maternal deaths, thereby bringing the nation closer to achieving Sustainable Development Goal 3.1 by 2030.

### 5.1 Study Limitations

A notable study limitation was the geographic restriction to three states in Nigeria, which was due to the pre‐designed implementation strategy. This setting potentially constrains the applicability of the findings to broader regions and countries, with diverse socioeconomic and politico‐administrative contexts. Additionally, the study is subject to time constraints, and the inability to capture the long‐term effects of the proposed interventions on the supply chain for uterotonics in Nigeria may impact the comprehensive understanding of the intervention's sustained impact over an extended period.

## Ethics Statement

This study was conducted in strict adherence to ethical guidelines and principles. Prior to the commencement of the research, ethical approval was obtained from the National Health Research Ethics Committee of Nigeria (NHREC Protocol Number NHREC/01/01/2007‐18/10/2021). All participants provided informed consent, ensuring they were fully aware of the study's aims, procedures, potential risks and benefits.

## Conflicts of Interest

The authors declare no conflicts of interest.

## Data Availability

The data that support the findings of this study are available on request from the corresponding author. The data are not publicly available due to privacy or ethical restrictions.
